# Alkalies a Remedy in Cutaneous Diseases

**Published:** 1846-09

**Authors:** 


### SELECTIONS

FROM

### AMERICAN AND FOREIGN JOURNALS.

---

*Alkalies as a remedy in Cutaneous Diseases.*—M. Devergie has recently published some interesting observations on the alkaline treatment of skin diseases. He has employed alkalies in both papular and scaly affections; but with most success in the former, and particularly in the various forms of lichen. He employs three salts: the bicarbonate of soda, the carbonate of soda, and the carbonate of potash. The first of these he administers only internally, and usually prescribes it in solution, in some mild stimulant bitter infusion, or in carbonic acid water, the latter being an imitation of Vichy water. The dose at first is fifteen grains daily, in three or four glasses of the infusion, and this dose is augmented by eight grains every third day, until it arrives at one drachm, which dose is not exceeded. Externally the alkaline treatment is used in four different forms: in baths, in lotions, in powder, and in ointment. For the preparation of baths, either the carbonate of soda or carbonate of potash is employed—the quantity used for a single bath varying from eight to sixteen ounces, the strength being gradually increased. For scrofulous or debilitated individuals, he recommends the addition of one pound of common salt to each bath. The alkaline lotions are found of special benefit in skin diseases affecting parts covered with hair, as in the scalp, where they are usually so obstinate. For a lotion, from two to three drachms of carbonate of soda are dissolved in a pint of water. To the benefits derivable from the use of this alkaline wash in chronic eczema and impetigo of the scalp, we can bear testimony, from an extensive experience of its employment, both in hospitals and in private practice. The alkalies are used in the form of powder, as a depilatory, in tinea and in scycosis mentis. M. Devergie, however, employs the alkalies chiefly in the form of ointment, and sometimes combines a little quicklime or a little sulphur, with them. He uses ointments of different strength, according to the nature of the disease. Thus, for lichen and its forms, the proportion is from eight to fifteen grains of carbonate of soda to the ounce of lard; for lepra, psoriasis, or icthyosis, fifteen to thirty grains to the ounce of lard; and for porrigo favosa, thirty to sixty grains, with a grain or two of quick-lime. It must be remembered, that the carbonate of potash is more caustic than the carbonate of soda. The following are some of the formulæ he employs:—*Alkaline Liniment.*—Carbonate of soda, ℥i.; olive oil, ℥iv.; the yolk of one egg; first moisten the carbonate of soda, and then incorporate it with the oil and yolk. *Alkaline Syrup.*—Bicarbonate of soda, ℥ss.; simple syrup, ℥viii.; dose, a tea-spoonful, morning and evening, in a glass of water. *Alkaline Powder.*—Carbonate of soda, in an impalpable powder, one part; fine starch ten parts. For external use only.—*Annuaire de Thérapeutique*, 1846.—*Bul. Med. Sci.*

